# Enhancing DASH Diet Adherence: A Cultural and Resource-Tailored Approach for African American Women

**DOI:** 10.1093/geroni/igaf122.2891

**Published:** 2025-12-31

**Authors:** Angela Groves, Yasir Mehmood

**Affiliations:** Western Michigan University, Portage, Michigan, United States; Michigan State University, East Lansing, Michigan, United States

## Abstract

Hypertension disproportionately affects African American women, placing them at a heightened risk for cardiovascular disease, kidney disease, and stroke. The Dietary Approaches to Stop Hypertension (DASH) diet lowers blood pressure, yet adherence is challenged by cultural food preferences, limited access to healthy foods, and affordability concerns. This study aimed to develop a culturally tailored DASH diet manual by engaging midlife and older African American women with hypertension to identify their food preferences, dietary practices, and available resources. Using a single-category focus group design, we recruited 11 participants from a church congregation in the Southwest United States. Key findings revealed six major themes and four subthemes influencing DASH diet adherence: (1) perspectives on diet, emphasizing sodium reduction and balanced nutrition; (2) awareness and education needs, including subthemes on (a) food label literacy and cooking skills and (b) tailoring materials for different reading levels; (3) community disparities in food access, with subthemes on (a) affordability challenges and (b) thrifty nutrition strategies; (4) challenges of Southern cooking traditions; (5) home appliance diversity in meal preparation; and (6) tailoring the DASH manual to dietary needs, including vegetarian and lactose-intolerant adaptations. Findings underscore the need for culturally sensitive interventions that integrate practical cooking skills, visual aids, and community-based support to improve adherence. Future research should explore culturally tailored DASH education programs, peer support models, and digital tools to enhance long-term adherence and blood pressure control among midlife and older African American women.

